# Unusual Foreign Body Found on Colonoscopy in an Adolescent Girl

**DOI:** 10.14309/crj.0000000000000762

**Published:** 2022-05-12

**Authors:** Charles B. Chen, Amala J. Alenchery, Lori Mahajan

**Affiliations:** 1 Department of Pediatric Gastroenterology, Hepatology, and Nutrition, Cleveland Clinic Foundation, Cleveland, OH; 2 Department of Pediatrics, Cleveland Clinic Foundation, Cleveland, OH

## CASE REPORT

A 13-year-old adolescent girl with a history of autism spectrum disorder presented with a 3-month history of intermittent, generalized abdominal pain and gagging. She had a history of constipation and had been evaluated 6 years previously for abdominal pain and weight loss. At that time, upper endoscopy and colonoscopy were performed to evaluate for inflammatory bowel disease. Both endoscopy and colonoscopy were visually normal; however, biopsies showed focal active inflammation in the ileum, transverse colon, ascending colon, and rectum. She was subsequently lost to follow-up.

**Figure 1.**
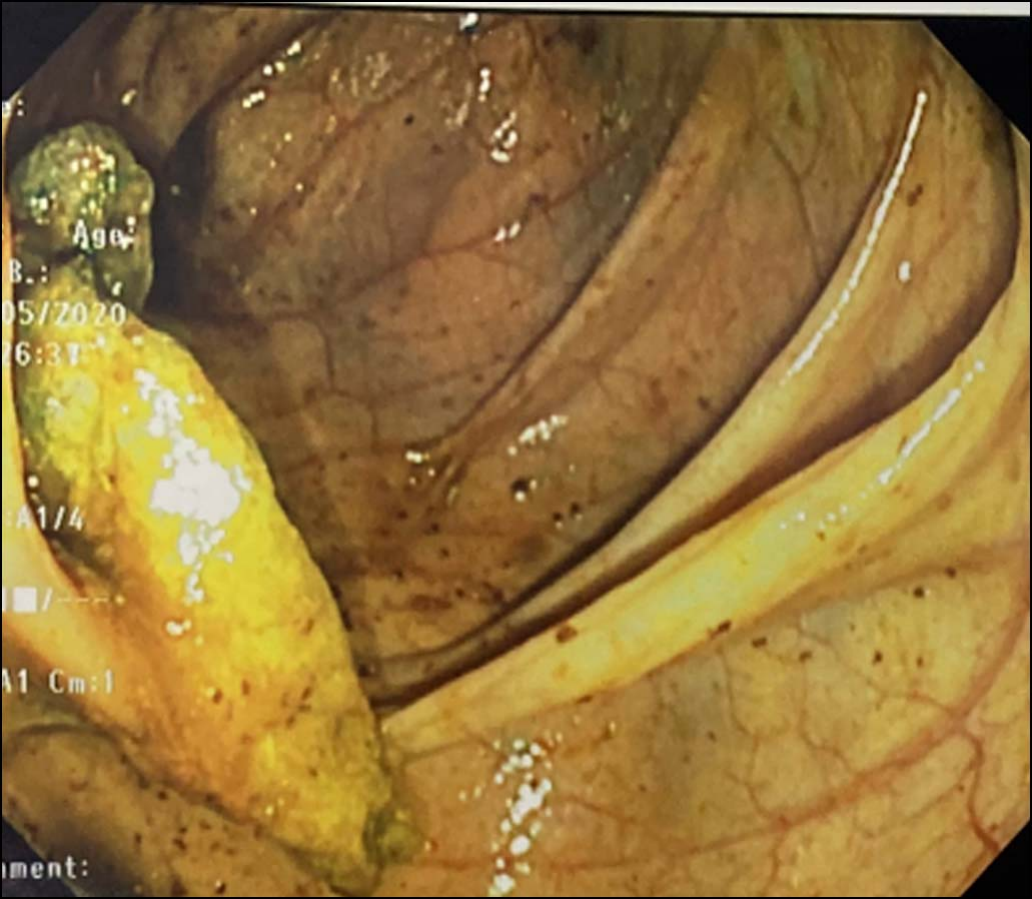
Foreign body (female hygiene product) seen on colonoscopy and spanning the IC valve. IC, ileocecal.

**Figure 2.**
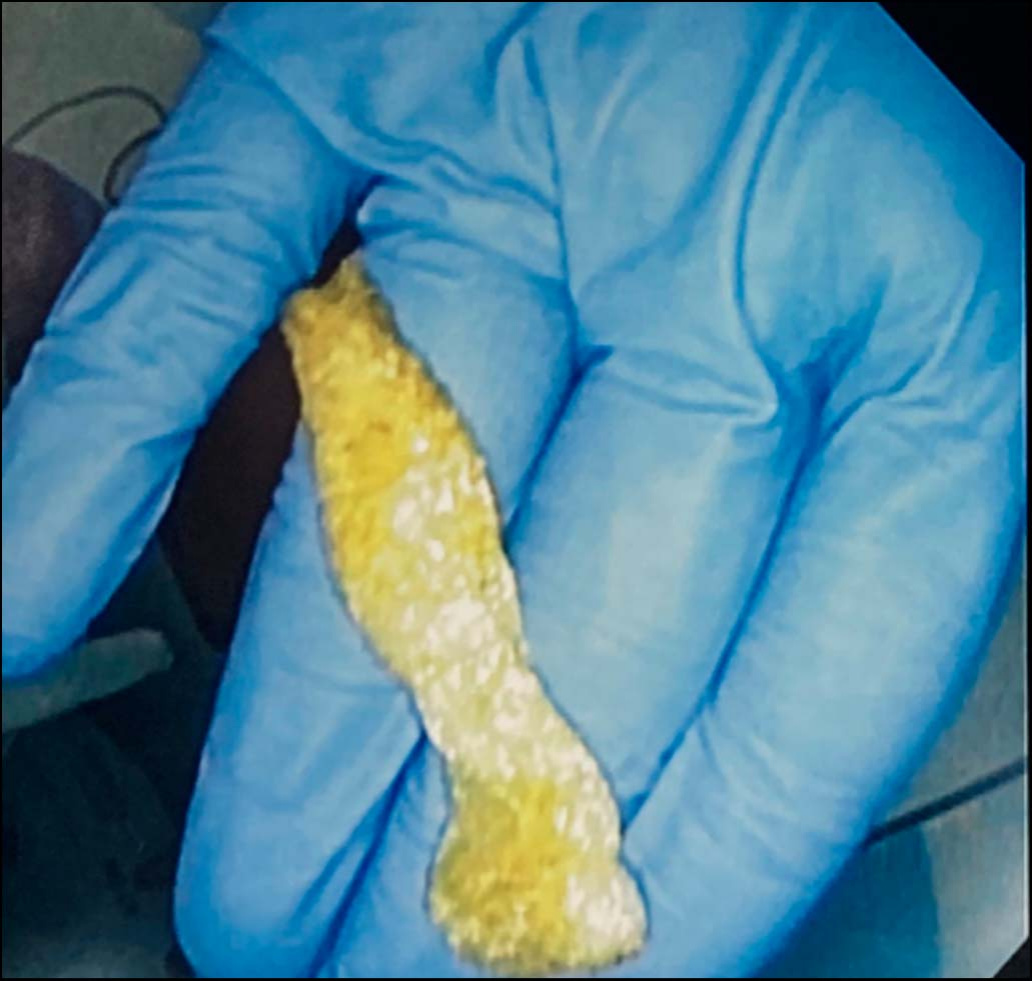
Female hygiene product removed.

She was nonverbal and developmentally delayed; however, the rest of her physical examination was unremarkable. Laboratory evaluation revealed thrombocytosis but otherwise normal complete blood count, complete metabolic panel, inflammatory markers, and fecal calprotectin. Despite optimizing her bowel regimen, she continued to have persistent abdominal pain and gagging. The duration of her symptoms and previous histologic findings raised concern for inflammatory bowel disease, and she underwent repeat upper endoscopy and colonoscopy. This time, a foreign body spanning the ileocecal valve was found that appeared to be a sponge (Figure 1). This was removed with biopsy forceps (Figure 2), and on closer inspection, the object appeared to be a tampon. When given this information, the mother recalled finding an opened tampon pack several months earlier. She stated that her daughter likely believed the package contained candy and must have consumed the contents. The rest of her endoscopy and colonoscopy was normal. After foreign body removal, her abdominal pain improved and her gagging resolved.

Foreign body ingestion occurs in more than 100,000 patients annually in the United States.^1^ Up to 20% of mentally handicapped adults demonstrate pica, which increases the risk of foreign body ingestion.^2^ Although many foreign bodies pass without difficulty, large or irregularly shaped objects have higher rates of impaction. The clinical presentation may be quite variable in patients with autism spectrum disorder, and communication barriers with healthcare providers make establishing a timely diagnosis challenging. Patients with neurodevelopmental disabilities may also have altered pain sensation, and the degree of pain or discomfort may be underappreciated.^2^ Large objects may warrant endoscopic removal or surgical evaluation especially if associated symptoms are present. In particular, the pyloric sphincter and the ileocecal valve are potential sites of impaction, as was seen in this patient.^3^ For healthcare providers, it is important to maintain a high index of suspicion for foreign body ingestion in patients with developmental delay who present with common gastrointestinal symptoms.

## DISCLOSURES

Author contributions: CB Chen, AJ Alenchery, and L. Mahajan made substantial contributions to the conception of this work as well as analysis and interpretation of important intellectual content. All authors participated in the review, drafting, and final approval of the manuscript. L. Mahajan is the article guarantor.

Financial disclosures: None to report.

Informed consent was obtained for this case report.
